# Analysis of the Interaction Mechanism of the Risk Factors of Gas Explosions in Chinese Underground Coal Mines

**DOI:** 10.3390/ijerph19021002

**Published:** 2022-01-17

**Authors:** Jinjia Zhang, Yiping Zeng, Genserik Reniers, Jie Liu

**Affiliations:** 1 School of Public Administration, Northwest University, Xi’an 710069, China; 2 Department of Statistics and Data Science, Southern University of Science and Technology, Shenzhen 518000, China; ypzeng@mail.ustc.edu.cn; 3 Faculty of Applied Economic Sciences, University of Antwerp, 2000 Antwerp, Belgium; genserik.reniers@uantwerpen.be; 4 Faculty of Public Security and Emergency Management, Kunming University of Science and Technology, Kunming 650093, China; liujie2004@kust.edu.cn

**Keywords:** coal mine, gas explosion accidents, risk factors, interaction relationship, risk management, DEMATEL–ISM–MICMAC

## Abstract

Gas explosion accidents easily cause severe casualties in Chinese underground coal mines. Systematic analysis of accident causation is crucial for the prevention of gas explosions. This study identifies the representative risk factors of gas explosions and determines the interrelationship among these risk factors to highlight weak links and develop countermeasures. A total of 21 representative risk factors of gas explosions were identified through 128 case studies and front-line investigations. On this basis, a five-level hierarchical structure model of gas explosions was established to explore the complex interrelationships among the representative risk factors based on a combination of the Decision-Making Trial and Evaluation Laboratory (DEMATEL) and Interpretive Structural Modeling (ISM) methods. Moreover, the Matrix of Cross Impact Multiplications Applied to Classification (MICMAC) method was applied to achieve risk factor classification into four clusters, namely, driving factors, linkage factors, dependent factors and autonomous factors. The results indicated that the interrelationships and emergence properties among the risk factors may cause gas explosions, which should give more attention to the interrelationships among multiple factors and multiple subsystems for coal enterprises. Meanwhile, the complex geological conditions, poor safety supervision, inadequate safety education and training, incomplete execution safety regulations and poor safety technology and input are the long-term focus of risk management for coal enterprises. Finally, 10 countermeasures were proposed to control these representative risk factors and interrelationships. The results are helpful to the development of gas explosion risk management policies and to the preferential allocation of limited resources to resolve these issues.

## 1. Introduction

China is the largest country in terms of coal production and consumption, and coal is mainly used as power fuel. In 2020, 3.9 billion tons of raw coal was produced in China, and coal consumption accounted for 56.8% of the total energy consumption [1]. However, gas explosions are major accidents in Chinese underground coal mines. Methane is a key constituent of gas, and air and gas (when the concentration ranges from 5 to 15%) can form inflammable yet explosive mixtures [2]. From 2015 to 2019, 57 gas explosion accidents occurred in Chinese underground coal mines, resulting in 365 fatalities [3]. Gas explosions in underground coal mines have also occurred in other major coal-producing countries [2]. The most notable gas explosions are listed in Table 1. Considering these accidents, there exists an urgent need to identify the risk factors and enhance the risk management of gas explosions.

The coal mine production system is a complex multidimensional system that covers multifactor, multisubsystem and multispatial structures, which are complex, dynamic and nonlinear systems. Safety managers should understand these complex systems to identify risk factors and develop risk management policies. Previous studies have analyzed the causation of gas explosions from different perspectives, such as gas accumulation, ignition source and occurrence location [4,5,6,7,8]. Chen et al. [4] found that gas explosion accidents primarily resulted from the chaotic management of ventilation facilities and electromechanical equipment. Dursun [5] statistically analyzed methane explosions in Turkey from 2010 to 2017 and revealed that gas accidents accounted for 68.34% of the death toll. Düzgün and Leveson [6] systematically investigated the Soma Mine Disaster with the Causal Analysis based on Systems Theory (CAST) method and indicated that sociotechnical factors played a crucial role in the Soma Mine Disaster. Kurlenya and Skritsky [7] analyzed gas explosions in Kuzbass mines and argued that the major cause was spontaneous coal combustion in mined-out areas. Zhu et al. [8] found that approximately 55% of all gas explosions occurred in so-called low-gas emission rate coal mines and that 44% of gas explosions were related to central ventilation systems. Scholars have indicated that human factors (e.g., illegal production, skill-based errors and violations) play a leading role in gas explosion accidents [9,10,11,12]. Lenné et al. [9] and Patterson et al. [10] argued that skill-based errors are the most important cause of coal mine accidents. Yin et al. [11] analyzed violations and the distribution characteristics of gas explosions considering the occurrence location, operation process and equipment installation. Zhang et al. [12] determined that violating safety regulations and neglecting safety priorities are important causes of coal mine accidents. Other researchers have focused on the physical characteristics of gas explosions and risk assessment [13,14,15,16]. Demir et al. [13] studied the flame acceleration characteristics of compressible gas and revealed that gas compression moderated flame acceleration by the type of fuel, thermal-chemical parameters and object geometry. Kundu et al. [14] reported that a methane–air mixture explosion is very serious under turbulent flow field and high-ignition energy conditions. Mitu et al. [15] examined the propagation characteristics of methane–air mixtures diluted by inert gases and found that CO_2_ was the most effective inert additive. Pang et al. [16] analyzed the distribution characteristics of flame regions and key influencing factors in coal tunnels. Bagherpour et al. [17] assessed the safety risk in Iranian coal mines from the perspective of preventive–preparative measures and indicated that methane explosions, coal dust explosions and traffic accidents were the most hazardous. Kabanov et al. [18] proposed assessment guidelines and a model of the probability of miner injury upon methane–air explosion in coal mines considering the explosion distance, initial composition and excavation parameters. Li et al. [19] identified fan and electrical failures as the two major causes of gas explosions using fuzzy-AHP and Bayesian networks. Shi et al. [20] developed an improved IAHP identification model of the influencing factors of gas explosions based on the FT and IAHP methods. The aforementioned studies have contributed significantly to the prevention of gas explosions and have improved the cause analysis of coal mine accidents. However, these studies mainly focused on the role of unsafe behavior, equipment failures and physical characteristics of gas explosion accidents. Since coal mining entails a complex sociotechnical system involving human factors, equipment factors, environment factors and management factors, this system is unavoidably, directly or indirectly, affected by these factors. Interactions may include linear or nonlinear effects and may cause gas explosion accidents. An important issue is that the key risk factors of past gas explosions and their interactions were not adequately considered, and similar risk factor chains frequently occur. In the coal mining system, an in-depth understanding of the interaction mechanism of the risk factors of gas explosions is vital for safe production. The aforementioned is a prerequisite to prevent gas explosions and improve risk management. If safety managers cannot clearly identify the diversity and interactions among the risk factors of gas explosions from a system perspective, this can hinder the identification of weak points in the analysis of gas explosions and the development of countermeasures.

Gas explosions in the multidimensional coal mining system follow a complex process and mode, and rational models are helpful when addressing complex problems [21,22]. Suitable methods have been developed to analyze accident causation in complex system domains, such as the Structural Equation Model (SEM) [23], Petri Nets (PN) [24], Decision-Making Trial and Evaluation Laboratory (DEMATEL) [25], Bayesian Networks (BN) [26], Neural Networks (NN) [27], Interpretive Structural Modeling (ISM) [28] and Matrix of Cross Impact Multiplications Applied to Classification (MICMAC) [29]. The DEMATEL technique is an effective method to analyze the cause–effect relationships among complex system factors and to translate these relationships into a visual structural model using graph theory [25]. ISM establishes a visual structural map by decomposing a complex system into various factors and has been adopted to describe the hierarchical structure and relationship between multiple factors [28]. The MICMAC method can classify the driving and dependence power of the different factors to provide an in-depth understanding of the interrelationships among these factors. The combination in the DEMATEL–ISM–MICMAC method may be well suited to analyze the interaction among the risk factors of gas explosions.

This study aims to establish a systematic model capturing the risk factors of gas explosions and their interrelationships in Chinese coal mines by employing a combination of the DEMATEL and ISM methods. On this basis, the driving and dependence values of the risk factors of gas explosions are determined with the MICMAC method, and countermeasures are proposed to address these risk factors and complex interrelationships. This study can enhance the risk management of gas explosions and allocate high-priority resources to address the weak links in coal production.

## 2. Identification of the Representative Risk Factors of Gas Explosions

**Figure 1:**
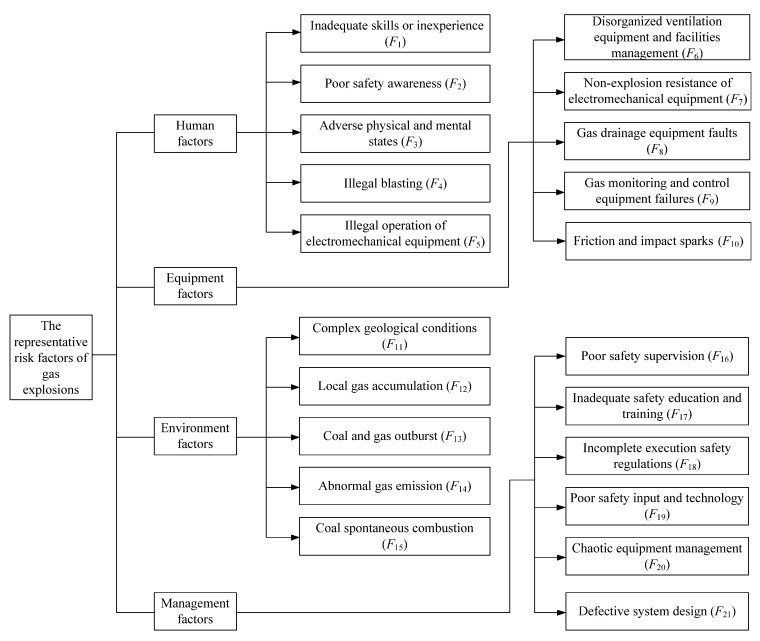
Index system of the representative risk factors of gas explosions.

The complexity of the coal mining system determines the causation complexity of gas explosions. According to the statistics of 128 extraordinarily severe gas explosion accidents (thirty or more fatalities in one accident) in Chinese coal mines from 1950 to 2019 [12,30], the accidents were the result of the interactions among multiple factors, including safety violations by miners, equipment faults, environment conditions and management errors. The 56 major risk factors in these 128 cases of extraordinarily severe gas explosions are presented in Table 2. Extraordinarily severe gas explosions usually occur under causation complexity and result in significant economic losses, numerous casualties and a serious social impact. Thus, these cases are associated with authoritative investigation reports and detailed causation analyses, providing suitable data and materials for this study. Moreover, we conducted several field investigations in Henan Province, Shanxi Province and Guizhou Province. In realistic coal production systems, the interactions among the risk factors were found to destabilize the system and increase the system fuzziness, thereby reducing the human capability of risk identification and increasing the frequency of accidents. Accident prevention usually focuses on human factors, equipment factors, environment factors and management factors [4,12,21]. Based on the above cause analysis and investigations in coal mines, we identified representative risk factors of gas explosions from the four aforementioned types of factors. In summary, an index system of the representative risk factors of gas explosions in Chinese coal mines was established, as shown in Figure 1.

### 2.1. Human-Related Representative Risk Factors

Safety violations and operation errors by miners are the predominant risk factors of gas explosions. However, human properties (e.g., physical states, work experience, cognition and awareness) are closely related to safety violations and operation errors, as determined through case analysis and individual interviews. Human-related representative risk factors are summarized in detail considering the following five aspects: inadequate skills or inexperience, poor safety awareness, adverse physical and mental states, illegal blasting and illegal operation of electromechanical equipment [9,10,11,12,30].

Inadequate skills or inexperience refers to unskilled or inexperienced professionals, respectively, in certain types of jobs, such as gas inspectors, blasters and electricians, which could easily lead to safety violations or operation errors in coal production.

Poor safety awareness suggests that miners exhibit poor safety cognition, safety values and safety attitudes, which translates into safety violations of operation procedures and safety regulations, such as reducing the gas inspection frequency, blasting with inadequately sealed holes and working in gas beyond the limitation area.

Adverse physical and mental states mainly indicate that miners suffer with poor health and exhibit a passive mental state, respectively, owing to social factors, family factors and poor operation environment, which affect their operational accuracy, safety awareness and reaction capacity.

Illegal blasting refers to the violating procedures and regulations of blasting, such as blasting without using a water stem, blasting with an inadequately sealed hole and bulldozing. Illegal blasting usually leads to flames in coal production, which is one of the most common ignition sources of gas explosions.

Illegal operation of electromechanical equipment refers to a situation wherein a special craft worker or miner violates the regulations concerning electromechanical operation, such as maintenance under power and bare cable joints. Illegal operation of electromechanical equipment easily causes an electric spark, which is one of the primary ignition sources of gas explosions.

### 2.2. Equipment-Related Representative Risk Factors

The reliability, explosion-resistance properties and proper operation of equipment, such as ventilation equipment, electric coal drills and cables, are important risk factors of gas explosions. Equipment unreliability or faults usually result in gas accumulation or electric sparks. Equipment-related representative risk factors were comprehensively analyzed from the following five perspectives: disorganized ventilation equipment and facilities management, non-explosion resistance of electromechanical equipment, gas drainage equipment faults, gas monitoring and control equipment failures and friction and impact sparks [4,8,12,30].

Disorganized ventilation equipment and facilities management primarily refers to the improper installation and operation of ventilation equipment, damaged ventilation equipment and the untimely maintenance of ventilation facilities. Examples include incorrect local fan installation, casually turning on and off local fan power and the untimely maintenance of damaged ventilation ducts, which are the primary causes of gas accumulation.

Non-explosion resistance of electromechanical equipment pertains to the loss of the explosion resistance properties of outer shells or parts of electromechanical equipment, such as electric coal drills, signal devices, junction boxes and cables, which represent the most common ignition sources.

Gas drainage equipment faults mainly refer to faults in drills, gas pumps, drainage pipes and orifice flowmeters, which impact gas drainage and the absolute gas emission quantity. Gas drainage equipment faults are mainly attributable to violations by miners, falling rocks, the overloaded operation of equipment and untimely maintenance.

Gas monitoring and control equipment failures refer to the damaged components and parts owing to external causes, such as the illegal operation triggering of signal interrupts, falling rocks damaging communication cables and a humid environment causing a false alarm in methane sensors. Damage to these components results in a failure to issue alerts when gas concentrations exceed the safety limit, a failure to detect local gas accumulation and a failure to shut off power.

Friction and impact sparks largely refer to the sparks produced by rock and ironwork due to friction and impact, respectively. These sparks are difficult to prevent and comprise a major ignition source of gas explosions. Examples include friction sparks originating from wire rope hoists, impact sparks stemming from rock and steel brackets and impact sparks due to falling rocks.

### 2.3. Environment-Related Representative Risk Factors

The complex environment of an underground coal mine provides a hazard-formative hotbed of gas explosions. Abrupt changes in geological conditions usually increase the methane concentration within the operation environment. Environment-related representative risk factors were analyzed regarding the following five aspects: complex geological conditions, local gas accumulation, coal and gas outburst, abnormal gas emission and spontaneous coal combustion [2,3,5,7,8,12,30].

Complex geological conditions include the gas content in coal seams, roof stability, geologic faults and periods of spontaneous combustion, which might result in coal and gas outbursts, abnormal gas emissions, spontaneous coal combustion, etc.

Local gas accumulation corresponds to reaching a methane gas concentration of 2% within a space greater than 0.5 m^3^ in an underground coal mine. Local gas accumulation usually occurs in heading faces, return airways and upper corners because of the chaotic management of local fans or ventilation ducts.

A coal and gas outburst is a complicated and dynamic phenomenon in which large amounts of coal and gas, respectively, suddenly erupt from coal rocks in a very short space of time during mining. These outbursts easily cause secondary disasters, such as gas explosions.

Abnormal gas emission is the phenomenon of an abruptly increasing gas concentration, which results in a sudden eruption of large amounts of gas from an underground local area during mining. Abnormal gas emission is difficult to control and can easily result in gas explosions when gas encounters an ignition source.

Spontaneous coal combustion is the phenomenon in which coal oxidation generates heat exceeding the self-ignition point of coal and is a major ignition source of gas explosions in the goaf. For example, breakages or cracks in a sealed wall can trigger the spontaneous combustion of residual coal in the goaf.

### 2.4. Management-Related Representative Risk Factors

Management errors constitute the underpinning mechanism of gas explosions. These errors are more notably related to concealment and complexity. Management errors are the underlying cause of accidents [21]. Management errors indirectly cause safety violations and affect the implementation of safety regulations and measures. Management-related representative risk factors were explored from the following six perspectives: poor safety supervision, inadequate safety education and training, incomplete execution safety regulations, poor safety input and technology, chaotic equipment management and defective system design [4,6,12,21,22,30].

Poor safety supervision indicates an inadequate safety inspection to identify human errors and equipment faults, as well as the poor execution of safety regulations and measures. These factors greatly affect the behavior and safety attitude of miners and the reliability of the equipment.

Inadequate safety education and training refers to situations where the safety training period is insufficiently long, an outdated training method is applied, training lacks pertinence or the training content is separated from practice. This impacts the operating skill, safety knowledge and safety awareness of miners.

The incomplete execution of safety regulations reflects coal enterprises and miners not completely following rules and regulations, operation procedures, prevention measures and requirements for installation equipment in regard to safety regulations.

Poor safety input and technology are mainly revealed in outdated safety technology and equipment and a lack of capital investment in safety facilities, safety equipment, safety education and training, labor protection appliances and accident rescue and prevention measures. These conditions may affect miners’ operation and hinder the development of technology and equipment for gas treatment in coal mines.

Chaotic equipment management primarily suggests the incorrect equipment installation, the illegal operation of equipment, the use of faulty equipment and an inadequate amount of equipment. Examples include the incorrect installation of local fans, the maintenance of equipment under power, an inadequate drill number and the untimely maintenance of electric coal drills without explosion resistance. These situations indirectly cause gas accumulation and electric sparks.

A defective system design is primarily shown in the unreasonable design of ventilation systems and the ineffective design of gas drainage systems. Examples include series ventilation, unreasonable drill hole spacing and inadequate hole depths, which indirectly result in gas accumulation and abnormal gas emissions.

## 3. Methodology

**Figure 2:**
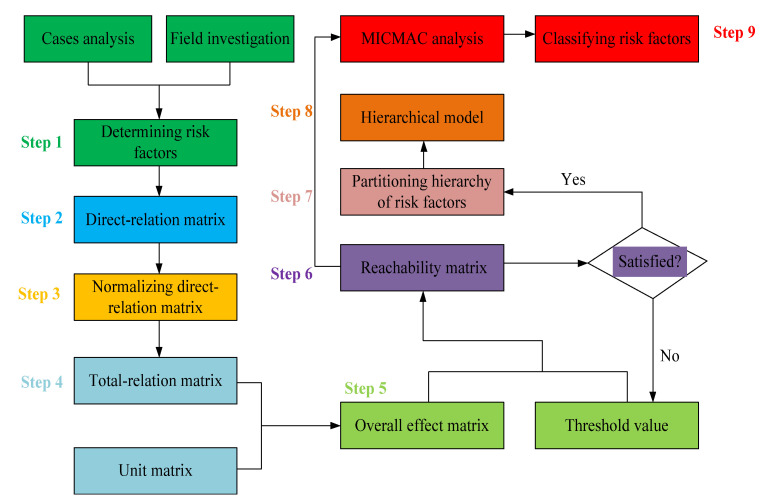
The flowchart of the established hierarchical structure model of gas explosions.

Although the DEMATEL, ISM and MICMAC models are effective tools to analyze the complex relationships among factors within a complex system, the advantage of the integrated DEMATEL–ISM–MICMAC method lies in the complementarity of the three individual models. The ISM model explains the macroscopic hierarchical structure and interaction relationships among the risk factors of gas explosions, but the calculations of the reachability matrix can be quite complex. The DEMATEL model can simplify the calculation process of the reachability matrix in the ISM method. Moreover, MICMAC analysis classifies the driving and dependence power among the risk factors based on the reachability matrix of the ISM method [29]. Therefore, an integrated DEMATEL–ISM–MICMAC method is effective in systematically describing the interrelationships among the risk factors of gas explosions. A hierarchical structure model of gas explosions was established, of which the flowchart is shown in Figure 2.

Based on the principle and procedures of DEMATEL and ISM, the integrated DEMATEL–ISM–MICMAC method is briefly summarized as follows:

Step 1: Determining representative risk factors. The 21 representative risk factors of gas explosions are determined based on 128 case analyses and field investigations, as shown in Figure 1.

Step 2: Constructing the direct-relation matrix. We assign five-level scores to the pair-wise comparison scale of the representative risk factors as follows: 0 (no influence), 1 (low influence), 2 (medium influence), 3 (high influence) and 4 (extremely high influence). The comparative result between the risk factors is established in the non-negative direct-relation matrix *F* = [*f_ij_*]*_n_*_×*n*_, in which *f_ij_* refers to the degree that risk factor *i* affects risk factor *j*; and when *i* = *j*, the diagonal risk factor *f_ij_* = 0. The seven experts in Table S1 were repeatedly asked to assess the relationships among the 21 representative risk factors of gas explosions. The experts determined the pairwise comparison results of the 21 representative risk factors by responding to the following question: “Do you believe factor *F_i_* directly affects factor *F_j_*? If so, please characterize the relationship on a scale from 0 to 4”. As expected, the different experts provided different opinions regarding the relationship between any two factors. Therefore, the principle stating that the minority is subordinate to the majority was applied to conclude the assessment. Thus, the relationships among the 21 representative risk factors were established as a direct-relation matrix, as summarized in Table S2.

Step 3: Normalizing the initial direct-relation matrix. According to the direct-relation matrix *F* = [*f_ij_*]*_n_*_×*n*_, the normalized direct-relation matrix *D* can be calculated with Equation (1). All representative risk factor values in matrix *D* range from 0 to 1, and the main diagonal representative risk factors are equal to 0.

(1) $$D=\frac{1}{\underset{1\leq i\leq n}{\max}\sum_{j=1}^{n}f_{ij}}F$$

Step 4: Obtaining the total-relation matrix. The total-relation matrix *T* can be obtained with Equation (2) when the normalized direct-relation matrix *D* is determined. In this equation, *I* denotes the unit matrix, and *t_ij_* denotes the elements of the total-relation matrix *T*.

(2) $$T=D{\left(I-D\right)}^{-1}={\left[t_{ij}\right]}_{n\times n}$$

Step 5: Establishing the overall effect matrix *H*. The total-relation matrix *T* only reflects the interactive relationship and correlation degree between the different representative risk factors rather than revealing the impact of each factor. Hence, the overall effect matrix *H* is calculated with Equation (3):

(3) $$H=T+I={\left[h_{ij}\right]}_{n\times n}$$

where *I* is the unit matrix, and *h_ij_* denotes the direct and indirect influence degrees of factor *i* on factor *j*.

Step 6: Determining the reachability matrix *U*. According to the overall effect matrix *H*, the reachability matrix *U =* [*u_ij_*]*_n_*_×*n*_ can be determined with Equations (4) and (5):

(4) $$u_{ij}=\{\begin{array}{c} \begin{array}{cc} 1， & h_{ij}\geq \lambda \end{array}\\ \begin{array}{cc} 0, & h_{ij}\leq \lambda \end{array} \end{array}$$

(5) λ=α+β

where *u_ij_* indicates whether factor *i* affects factor *j* under the given threshold *λ*. *h_ij_* > *λ* indicates the presence of influence and *u_ij_* = 1; *h_ij_* ≤ *λ* indicates no influence, and *u_ij_* = 0. *α* and *β* are the average and standard deviation, respectively, of the factors of the total-relation matrix *T* [31].

Step 7: Partitioning the level of representative risk factors is performed according to the following steps:

The reachability set (*R_i_*) includes the factors equivalent to the columns with a value of 1 in the *i*-th row of the reachability matrix *U*, whereas the antecedent set (*A_i_*) includes the factors equivalent to the rows with a value of 1 in the *i*-th column of the reachability matrix *U*. The intersection set (*R_i_* ∩ *A_i_*) includes factors in both reachability and antecedent sets.

The reachability set (*R_i_*) and antecedent set (*A_i_*) are denoted respectively as follows:

(6) $$R_{i}=\left\{u_{j}|u_{j}\in U,u_{ij}=1\right\},\left(i=1,2,\cdots ,n\right)$$

(7) $$A_{i}=\left\{u_{j}|u_{j}\in U,u_{ji}=1\right\},\left(i=1,2,\cdots ,n\right)$$

Step 7.1: The risk factors are partitioned in Level I as follows:

(8) $$R_{i}=R_{i}\cap A_{i}\;$$

Step 7.2: The Level I risk factors are separated from *R_i_*, *A_i_* and *R_i_* ∩ *A_i_*, and the Level II risk factors are searched, similar to Step 7.1.

Step 7.3: Step 7.2 is repeated until the level of each risk factor is identified.

Step 8: An ISM diagram of gas explosions is created.

An ISM diagram of gas explosions is created through the level partitioning results of the representative risk factors. First, the Level I risk factors are placed at the top of the hierarchical structure diagram. The Level II risk factors are placed at the second position of the hierarchical structure diagram, etc., until the risk factors at the last level are placed at the lowest position in the hierarchical structure diagram.

Step 9: MICMAC analysis.

The driving and dependence power values in MICMAC analysis can be calculated with Equations (9) and (10), respectively, based on the reachability matrix *U* of the ISM method.

(9) $$DR_{i}=\sum_{j=1}^{n}u_{ij},i=1,\;2,\;\cdots ,\;n$$

(10) $$DE_{j}=\sum_{i=1}^{n}u_{ij},j=1,\;2,\;\cdots ,\;n$$

where *DR_i_* is the driving power of risk factor *i*, and *DE_j_* is the dependence power of risk factor *j*. MICMAC analysis divides the risk factors into the following four categories: autonomous factors with low driving and dependence power values, dependent factors with low driving power and high dependence power values, linkage factors with high driving and dependence power values and independent factors with high driving power and low dependence power values [32].

## 4. Results

**Figure 3:**
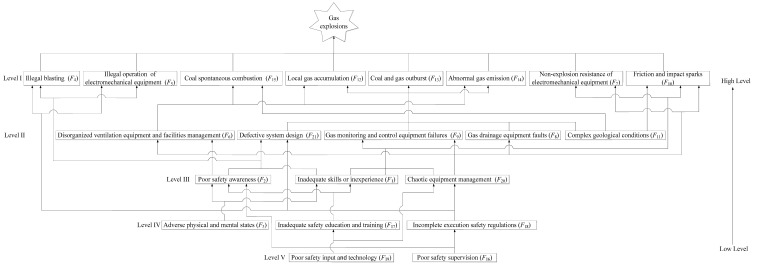
The hierarchical structure model of the representative risk factors of gas explosions.

**Figure 4:**
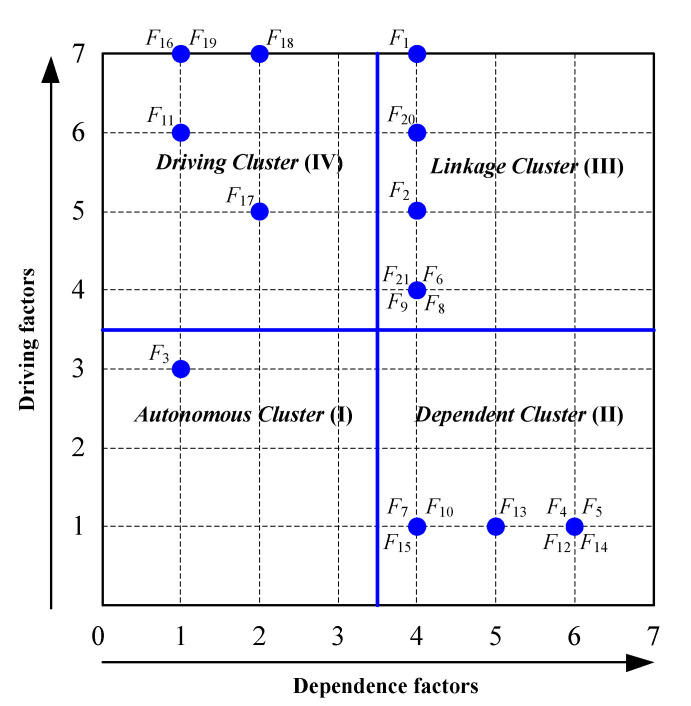
An MICMAC diagram of the representative of gas explosions.

In steps one and two of the DEMATEL–ISM–MICMAC method, the relationships among the 21 representative risk factors were established as a direct-relation matrix, as summarized in Table S2. Similarly, by following steps three and four, the total-relation matrix *T* of the representative risk factors was calculated with Equations (1) and (2), as listed in Table S3. Moreover, by following step five, the overall effect matrix *H* of the representative risk factors was obtained with Equation (3), as presented in Table S4. Following step six, *λ* was set to 0.0095 via Equation (5), and the reachability matrix was determined with Equations (4) and (5), as listed in Table S5. Based on the principle of step seven, the results of level partitioning of the representative risk factors of gas explosions were obtained with Equations (6) and (8), as presented in Table S6. According to step eight, a hierarchical structure model of the representative risk factors of gas explosions was constructed, as shown in Figure 3. The driving and dependence power values of the representative risk factors of gas explosions were calculated with Equations (9) and (10), respectively, based on step nine and the reachability matrix. The driving-dependence power figure is shown in Figure 4.

As is shown in Figure 3, the 21 representative risk factors of gas explosions can be divided into five levels, which clearly reflect the hierarchical structure of the representative risk factors and their interaction mechanisms. Illegal blasting (*F*_4_), the illegal operation of electromechanical equipment (*F*_5_), the non-explosion resistance of electromechanical equipment (*F*_7_), friction and impact sparks (*F*_10_), local gas accumulation (*F*_12_), coal and gas outburst (*F*_13_), abnormal gas emission (*F*_14_) and spontaneous coal combustion (*F*_15_) were located at the top level (Level I). These factors cannot influence the other representative risk factors and are easily perceived in causation analysis of gas explosions; therefore, these factors are regarded as direct causes. Inadequate skills or inexperience (*F*_1_), poor safety awareness (*F*_2_), disorganized ventilation equipment and facilities management (*F*_6_), gas drainage equipment faults (*F*_8_), gas monitoring and control equipment failure (*F*_9_), complex geological conditions (*F*_11_), chaotic equipment management (*F*_20_) and defective system design (*F*_21_) are located at the middle level (Levels II and III). These factors both affect and are affected by other representative risk factors. Therefore, these factors were considered as connective factors and indirect causes of gas explosions in this study. Adverse physical and mental states (*F*_3_), poor safety supervision (*F*_16_), inadequate safety education and training (*F*_17_), incomplete execution safety regulations (*F*_18_) and poor safety input and technology (*F*_19_) were located at the bottom two levels (Levels IV and V); these factors not only affect other representative risk factors usually but also hardly exert their influence on the coal production and accident analysis. Hence, these factors were considered the depth factors and comprised the root cause.

In MICMAC analysis, the representative risk factors of gas explosions were grouped into four clusters based on their driving and dependence power values, as shown in Figure 4. The representative risk factors of gas explosions of *F*_11_, *F*_16_, *F*_17_, *F*_18_ and *F*_19_ attained high driving power but low dependence power values and were located in a driving cluster (IV). Thus, these factors are the most fundamental (but often overlooked) risk factors from an overall network perspective of gas explosions. These factors must be developed in risk management systems as fundamental tasks. The representative risk factors of gas explosions of *F*_1_, *F*_2_, *F*_6_, *F*_8_, *F*_9_, *F*_20_ and *F*_21_ attained high driving and dependence power values and were located in linkage cluster (III). Thus, *F*_1_, *F*_2_, *F*_6_, *F*_8_, *F*_9_, *F*_20_ and *F*_21_ are risk paths from an evolution network perspective of gas explosions. These factors should be addressed with targeted measures to reduce their occurrence probability. The representative risk factors of gas explosions of *F*_4_, *F*_5_, *F*_7_, *F*_10_ *F*_12_, *F*_13_, *F*_14_ and *F*_15_ attained low driving power but high dependence power values and were located in dependent cluster (II). Therefore, these factors are the most concrete risk factors in coal production. These risk factors should be identified and addressed in a timely manner during coal production. The representative risk factor *F*_3_ of gas explosions attained both low driving and dependence power values and was located in autonomous cluster (I). *F*_3_ is seemingly detached from the coal production system.

## 5. Discussion

In this study, a five-level hierarchical structure model of gas explosions was established using an integrated DEMATEL–ISM–MICMAC method, which provided a visualization of the interaction relationship among the representative risk factors. Not only were the interrelationships among the risk factors of gas explosions identified, but the categories of these interrelationships were also classified based on driving and dependence power values, such as driving factors, linkage factors, dependent factors and autonomous factors. Although existing systematic accident models, such as Accimap [33] and system theoretic accident modeling and process model (STAMP) [21], have described the accident causes from the systematic perspective, they still cannot fully present the interrelationships, categories and importance among accident factors. Similarly, compared to the statistical approach [4,8,11,12] and fuzzy mathematics [19,20], the proposed risk analysis model of gas explosions can establish a risk factor interactive network and account for the extent to which risk factors exert influence or are affected, which is helpful to address the complex interdependence among the risk factors of gas explosions.

**Figure 5:**
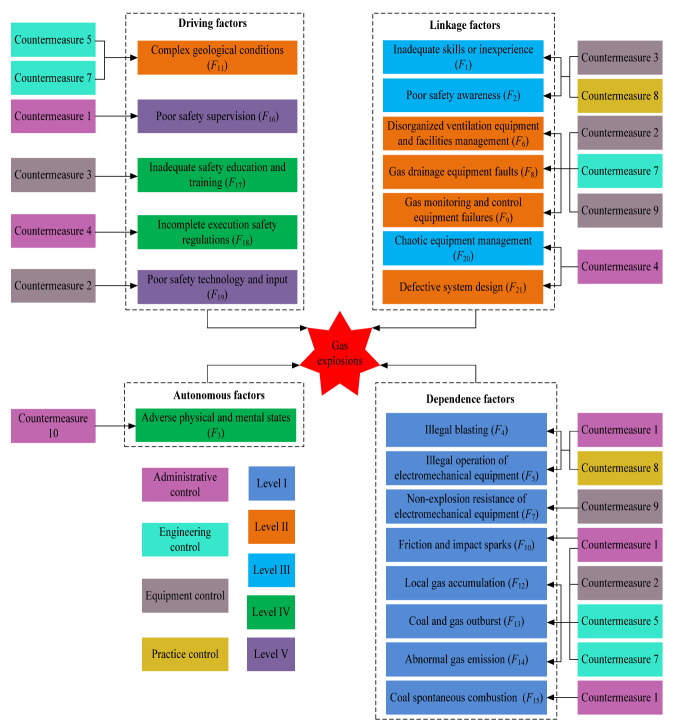
The representative risk factors and countermeasures.

Interestingly, we found that gas explosions result from the interactions and emergence properties among the risk factors, which gives more attention to the interrelationships among the multiple factors and multiple subsystems in coal production rather than only emphasizing the direct risk factors or causes. Gas explosion accidents should not simply be ascribed to human errors or equipment faults because a small risk factor might be converted into a substantial threat to safe production through interactions, but the emerging networks of gas explosions are usually overlooked. It can be found from Figure 3 and Figure 4 that complex geological conditions (*F*_11_), poor safety supervision (*F*_16_), inadequate safety education and training (*F*_17_), incomplete execution safety regulations (*F*_18_) and poor safety technology and input (*F*_19_) are the driving factors of gas explosions. Complex geological conditions (*F*_11_), such as gas pressure, gas content, low-permeability coal seams and spontaneous coal combustion tendency, directly affect local gas accumulation, abnormal gas emission, a coal and gas outburst and coal spontaneous combustion. Poor safety supervision (*F*_16_) directly induces a poor safety awareness and incomplete execution safety regulations among miners, even the miners’ safety violations. Inadequate safety education and training (*F*_17_) directly influences the safety awareness and operation behavior of miners, which is a major cause of poor safety awareness and safety violations. The incomplete execution of safety regulations (*F*_18_) directly triggers safety violations by miners, a defective system design, and chaotic equipment management, such as outburst prevention measures, gas extraction design or violations of safety regulations, resulting in abnormal gas emissions or coal and gas outbursts. Poor safety technology and input (*F*_19_) directly affects investments in safety training and the purchase or maintenance of gas-related equipment. It should be noted that complex geological conditions (*F*_11_), poor safety supervision (*F*_16_) and inadequate safety education and training (*F*_17_) are often mentioned in existing studies [4,9,10,11,12], while incomplete execution safety regulations (*F*_18_) and poor safety technology and input (*F*_19_) are the most likely to be overlooked. Simultaneously, inadequate skills and inexperience (*F*_1_), poor safety awareness (*F*_2_), disorganized ventilation equipment and facilities management (*F*_6_), gas drainage equipment faults (*F*_8_), gas monitoring and control equipment failures (*F*_9_), chaotic equipment management (*F*_20_) and defective system design exhibit a sensitive nature, which influences the driving factors and transfers them into dependent factors. Coal enterprises should give more attention to controlling these risk factors to reduce the risk transference from driving factors. For example, strengthening the safety awareness and professional skills among miners can reduce the influence of poor safety supervision and contribute to the prevention of safety violations (e.g., illegal blasting and operation under power). Compared to the linkage factors, adverse physical and mental states (*F*_3_) represent an isolated risk factor that slightly influences the safety awareness and behavior of miners due to coal mines prohibiting work under adverse conditions. Moreover, we should note that the dependent factors (*F*_4_, *F*_5_, *F*_7_, *F*_10_, *F*_12_, *F*_13_, *F*_14_ and *F*_15_) are only affected linkage factors and driving factors, which are the direct causes of gas explosions. Coal enterprises should eliminate the dependent factors of gas explosions in a timely manner. However, according to the risk emergence mechanism of gas explosions shown in Figure 3 and Figure 4, dependent factors can recur in coal production if driving and linkage factors cannot be effectively controlled. Thus, the following countermeasures are proposed from the perspective of the safety control system (e.g., administrative control, engineering control, equipment control, practice control) based on the analysis results of the DEMATEL–ISM–MICMAC method, as shown in Figure 5.

Countermeasure 1: A double prevention mechanism should be established. The double prevention mechanism includes risk ranking management and safety hazard identification-governance. Risk is a core concern of double prevention mechanisms, which insist on the pre-prevention of gas explosions. A double prevention mechanism constructs a simplified, powerful and direct risk management system that focuses on accurately identifying risk and addressing safety hazards in a timely manner. The principle of the double prevention mechanism is to control risks before safety hazards occur and eliminate safety hazards before accidents ensue.

Countermeasure 2: Capital investments in gas-related equipment should be increased. Safety investments are usually considered to affect the economic benefit in certain Chinese coal mines and are, therefore, neglected. Coal enterprises should increase investments in beam-sensor gas monitoring systems, large-aperture directional drills, intelligent ventilation systems and equipment updates and maintenance. This could help enhance the ventilation stability, monitoring accuracy, extraction efficiency and equipment reliability.

Countermeasure 3: A training platform for VR simulations should be constructed. The main mode of safety training is lectures and case analysis by the safety manager, which may not effectively increase the safety awareness and professional skills of miners. A training platform for VR simulations is an interactive device, including safety hazard identification training, professional skills training, accident and rescue training simulations and self-rescue training, which contributes to enhancing the safety awareness, professional skills and rescue capacity of miners.

Countermeasure 4: The effective implementation of safety regulations should be guaranteed. The effective implementation of safety regulations can realize the expected security performance, but certain coal mines place more emphasis on the comprehensiveness of regulations rather than effective implementation. Therefore, managers and miners of coal enterprises must follow stringent safety regulations, especially those regarding safety inspection systems, safety technology measures, operational safety rules, safety reward and punishment systems, etc.

Countermeasure 5: Composite anti-reflection and gas extraction technology should be implemented. The low permeability, high gas content and high gas pressure in coal seams are major causes of a low gas extraction efficiency, which directly results in abnormal gas emissions and coal and gas outbursts in coal mining. According to different gas existence conditions, coal enterprises should strictly adopt hydraulic fracturing technology, pressure relief technology involving air blasting, drilling technology for large-diameter boreholes and gas extraction roadway technology, which contributes to gas pressure relief and gas extraction [34].

Countermeasure 6: Squad-based safety construction should be strengthened. The squad is the smallest organization and front-line work unit. Coal enterprises should attach importance to the definition of clear responsibilities and safety-related reward–punishment systems in terms of squad leaders, which can develop the safety awareness level and safety management capability of squad leaders. Moreover, the squad-based standardization and enhancement of operation procedures, operation systems and operation requirements are vital to improve safety awareness among miners and reduce violations in coal production.

Countermeasure 7: The ventilation system, gas monitoring system and gas drainage system should be optimized. The ventilation network, gas monitoring network and gas drainage network should be constantly optimized in terms of layout, construction and operation. Moreover, coal enterprises should strengthen key equipment during the investment, upgrading and transformation phases, such as local fans and corresponding intelligent control systems, gas monitoring and control systems and large deep-hole drilling rigs.

Countermeasure 8: The operation procedures of special task types should be standardized. Safety violations by miners (e.g., blasters, electricians and gas inspectors) in special types of work, such as blasting with no use of water stems, operation under power and gas inspection omission, constitute a major ignition source of gas accumulations resulting in gas explosions. Coal enterprises should emphasize the standardization of the workflows, operation procedures and safety requirements of special types of tasks to notably develop safety habits and operation standards. It is critical to reduce the occurrence probability of ignition sources or gas accumulation.

Countermeasure 9: Key equipment should be properly maintained. Coal enterprises must strengthen the management of key equipment to prevent gas accumulation and eliminate the ignition sources of gas explosions, such as local fans, electric coal drills, cables, ventilation tubes, gas sensors, drilling rigs and drainage pumps. Moreover, safety managers must attach great importance to the key equipment running status in special areas, such as coal faces and heading faces. Moreover, coal enterprises must implement regular and unscheduled checks of key equipment, which are vital for the prevention of gas accumulation and ignition sources.

Countermeasure 10: The team concern of the union should be strengthened. The reinforcement of job and family stress brings job burnout to staff, which reduces their job satisfaction and has a bad effect on employees. The union should fully carry out its functions, which includes setting up specialized departments of health management, psychological consultation and social relief. These departments contribute to miners’ health, family and job stress and enhance the miners’ job satisfaction and life happiness.

## 6. Conclusions

To obtain a comprehensive and accurate understanding of the mechanism of gas explosions, this study explored the interactions among the risk factors of gas explosions and their categories. The results suggested that the risk factors of gas explosions are interconnected and revealed the emerging characteristics. Obviously, these interrelationships and the characteristics emerging among the risk factors may cause gas explosions, namely, complex geological conditions, poor safety supervision, inadequate safety education and training, incomplete execution safety regulations and poor safety technology and input are the long-term focus of risk management for coal enterprises. Thus, chain-cutting countermeasures of gas explosions must be implemented in Chinese coal mines. From a safety production perspective, understanding the interrelationships among risk factors could help safety managers to develop effective policies, standards and regulations to prevent gas explosions in Chinese coal mines, as the priority of the 21 risk factors was clearly indicated.

The proposed DEMATEL–ISM–MICMAC method suffers certain disadvantages. First, subjective factors (such as expert experience) are involved in the selection of the representative risk factors and the determination of the evaluation direct-relation matrix. Moreover, the established model facilitates static risk analysis rather than dynamic analysis of the interaction among risk factors, which need to develop the dynamic model through the large amounts of data. Finally, the results of the DEMATEL–ISM–MICMAC model are qualitative analysis results, while the interaction intensity among the identified risk factors should be explored in the future. Thus, future research should consider dynamic methods, such as complex networks and system dynamics.

## Figures and Tables

**Table 1:** Notable gas explosions of major coal-producing countries after 2000 [2].

Country	Year	Mine	Fatalities
China	2004	Chenjiashan	166
	2005	Sunjiawan	214
Columbia	2011	La Preciosa	21
India	2006	Bhatdee	50
	2010	Anjan Hill	14
Poland	2006	Halemba	21
Russia	2007	Ulyanovskaya	108
	2010	Raspadskaya	66
	2021	Listvia Shnaya	52
Turkey	2010	Karadon	30
	2014	Soma	301
Ukraine	2007	Zasyadko	80
	2011	Suhodolskaya–Vostochnaya	19
U.S.	2006	Sago	12
	2010	Upper Big Branch	29

**Table 2:** The comprehensive risk factors based on 128 cases [12,30].

No.	Risk Factors	Frequency
1	Not addressing hazards in a timely manner	41
2	Special craft miner working without skilled training	32
3	Not strictly implementing gas inspecting regulations	26
4	Adventure production without implementing corrective measures	23
5	Working in risk area	21
6	No or inadequate implementation of measures of discharging gas	18
7	Local fan stalling due to power failure	18
8	No or inadequate implementation of measures of gas drainage	14
9	Gob area resulting in spontaneous combustion	14
10	Gas monitoring and control equipment fault	14
11	Blasting with inadequate sealed-hole	13
12	Cable breakage or bare cable joint	13
13	Installing local fan in wrong location resulting in air recirculation	13
14	Drilling machine count without matching with actual production	12
15	Series ventilation	11
16	Gob area gas	10
17	Dismantling and beating cap-lamp	9
18	Blasting with inadequate resistance line	9
19	Strike spark of metal material or equipment hitting metal	9
20	Gas drainage equipment fault	9
21	Ventilation duct without extending in the heading face	9
22	Electric coal drill with non-explosion resistance	8
23	Short circuit of ventilation	8
24	No or inadequate implementation of measures of outburst prevention	8
25	Casually turn on and off the local fan	7
26	Maintenance with power	7
27	Ventilation system with no or poor design	7
28	Blasting with the wires exposed to the air	6
29	Bulldozing	6
30	Blasting with no use of water-stem	5
31	Coal and gas outburst	5
32	Connecting blasting bus bar to wires directly	4
33	Smoking	4
34	Roof falling with abnormal gas-effusion	4
35	Ventilation duct disjunction	4
36	Trolley wire resulting in spark	3
37	Sealed wall breakage with gas leakage	3
38	Junction box with non-explosion resistance	3
39	Mining top coal with abnormal gas-effusion	3
40	Blind roadway gas	3
41	Unqualified electrical equipment entry to underground coal mine	3
42	Open fire	3
43	Uninstalled or being installed local fan	3
44	Ventilation duct crevasse with serious air leakage	3
45	Ventilation duct with serious press	3
46	One-time filling explosive with multiple blasting	2
47	Scraper conveyer with short circuit	2
48	Signal device with non-explosion	2
49	Friction spark of wire rope of hoist	2
50	Rock falling with breaking cable	2
51	Upper corner gas	2
52	Roadway section with serious blockage	2
53	Local fan with non-explosion resistance	1
54	Strike spark of rock falling	1
55	Welding spark	1
56	One local fan ventilating air to multiple locations	1

## Data Availability

The data presented in in this study are available upon request from the corresponding author.

## Supplementary Materials

The following are available online at https://www.mdpi.com/article/10.3390/ijerph19021002/s1, Table S1: The profiles of interviewees; Table S2: The direct-relation matrix of the representative risk factors of gas explosions; Table S3: The total-relation matrix of the representative risk factors of gas explosions; Table S4: The overall effect matrix of the representative risk factors of gas explosions; Table S5: The reachability matrix of the representative risk factors of gas explosions; Table S6: The results of the level partitions of the reachability matrix Iteration I to Iteration IV.
