# Supplementary material for: Analysis of the Interaction Mechanism of the Risk Factors of Gas Explosions in Chinese Underground Coal Mines
Source: Int J Environ Res Public Health. 2022 Jan 17;19(2):1002. doi: 10.3390/ijerph19021002 (PMC8775391; doi:10.3390/ijerph19021002)
Supplement: Supplementary file 1 [file ijerph-19-01002-s001.zip › ijerph-1521319-supplementary.pdf]

Table S1. The profiles of interviewees.

Experts	Work organization	Role	Experience	Interview method
Expert A	China University of Mining & Technology (Beijing)	Professor	21 years	Face-to-face
Expert B	China University of Geosciences (Beijing)	Professor	18 years	Face-to-face
Expert C	Liaoning Technical University	Professor	24 years	Email
Expert D	Zhengzhou Coal Industry (Group) Co., Ltd.	Senior Engineer	14 years	Face-to-face
Expert E	Jizhong Energy Group Co., Ltd.	Senior Engineer	17 years	Email
Expert F	Shanxi Coking Coal Group Co., Ltd.	Senior Engineer	11 years	Face-to-face
Expert G	Huayang New Material Technology Group Co., Ltd	Senior Engineer	13 years	Email

Table S2. The direct-relation matrix of the representative risk factors of gas explosions.

	F_1	F_2	F_3	F_4	F_5	F_6	F_7	F_8	F_9	F_{10}	F_{11}	F_{12}	F_{13}	F_{14}	F_{15}	F_{16}	F_{17}	F_{18}	F_{19}	F_{20}	F_{21}
F_1	0	0	0	2	2	0	2	0	2	2	0	0	0	0	0	0	0	0	0	0	2
F_2	0	0	0	3	3	2	0	0	0	0	0	0	0	0	0	0	0	0	0	0	2
F_3	2	2	0	0	0	0	0	0	0	0	0	0	0	0	0	0	0	0	0	0	0
F_4	0	0	0	0	0	0	0	0	0	0	0	0	0	0	0	0	0	0	0	0	0
F_5	0	0	0	0	0	0	0	0	0	0	0	0	0	0	0	0	0	0	0	0	0
F_6	0	0	0	0	0	0	0	0	0	0	0	3	0	2	2	0	0	0	0	0	0
F_7	0	0	0	0	0	0	0	0	0	0	0	0	0	0	0	0	0	0	0	0	0
F_8	0	0	0	0	0	0	0	0	0	0	0	2	2	2	0	0	0	0	0	0	0
F_9	0	0	0	0	0	0	0	0	0	0	0	2	2	2	0	0	0	0	0	0	0
F_{10}	0	0	0	0	0	0	0	0	0	0	0	0	0	0	0	0	0	0	0	0	0
F_{11}	0	0	0	0	0	0	0	0	0	2	0	2	4	3	2	0	0	0	0	0	0
F_{12}	0	0	0	0	0	0	0	0	0	0	0	0	0	0	0	0	0	0	0	0	0
F_{13}	0	0	0	0	0	0	0	0	0	0	0	0	0	0	0	0	0	0	0	0	0

F_{14}	0	0	0	0	0	0	0	0	0	0	0	0	0	0	0	0	0	0	0	0	0
F_{15}	0	0	0	0	0	0	0	0	0	0	0	0	0	0	0	0	0	0	0	0	0
F_{16}	0	4	0	4	4	3	0	0	0	0	0	0	0	0	0	0	0	2	0	3	0
F_{17}	2	4	0	3	3	0	0	0	0	0	0	0	0	0	0	0	0	0	0	0	0
F_{18}	0	0	0	4	3	0	3	2	0	0	0	0	0	0	0	0	0	0	0	2	2
F_{19}	2	0	0	0	0	0	0	2	2	0	0	0	0	0	2	0	2	0	0	2	0
F_{20}	0	0	0	0	0	4	4	3	2	2	0	0	0	0	0	0	0	0	0	0	0
F_{21}	0	0	0	0	0	0	0	0	0	0	0	2	2	2	0	0	0	0	0	0	0

Table S3. The total-relation matrix of the representative risk factors of gas explosions.

	F_1	F_2	F_3	F_4	F_5	F_6	F_7	F_8	F_9	F_{10}	F_{11}	F_{12}	F_{13}	F_{14}	F_{15}	F_{16}	F_{17}	F_{18}	F_{19}	F_{20}	F_{21}
F_1	0	0	0	0.01	0.01	0	0.01	0	0.01	0.01	0	0	0	0	0	0	0	0	0	0	0.01
F_2	0	0	0	0.0225	0.0225	0.01	0	0	0	0	0	0	0	0	0	0	0	0	0	0	0.01
F_3	0.01	0.01	0	0	0	0	0	0	0	0	0	0	0	0	0	0	0	0	0	0	0
F_4	0	0	0	0	0	0	0	0	0	0	0	0	0	0	0	0	0	0	0	0	0
F_5	0	0	0	0	0	0	0	0	0	0	0	0	0	0	0	0	0	0	0	0	0
F_6	0	0	0	0	0	0	0	0	0	0	0	0.0225	0	0.01	0.01	0	0	0	0	0	0
F_7	0	0	0	0	0	0	0	0	0	0	0	0	0	0	0	0	0	0	0	0	0
F_8	0	0	0	0	0	0	0	0	0	0	0	0.01	0.01	0.01	0	0	0	0	0	0	0
F_9	0	0	0	0	0	0	0	0	0	0	0	0.01	0.01	0.01	0	0	0	0	0	0	0
F_{10}	0	0	0	0	0	0	0	0	0	0	0	0	0	0	0	0	0	0	0	0	0
F_{11}	0	0	0	0	0	0	0	0	0	0.01	0	0.01	0.04	0.0225	0.01	0	0	0	0	0	0
F_{12}	0	0	0	0	0	0	0	0	0	0	0	0	0	0	0	0	0	0	0	0	0
F_{13}	0	0	0	0	0	0	0	0	0	0	0	0	0	0	0	0	0	0	0	0	0
F_{14}	0	0	0	0	0	0	0	0	0	0	0	0	0	0	0	0	0	0	0	0	0
F_{15}	0	0	0	0	0	0	0	0	0	0	0	0	0	0	0	0	0	0	0	0	0
F_{16}	0	0.04	0	0.05	0.049	0.0303	0	0	0	0	0	0	0	0	0	0	0	0.01	0	0.024	0
F_{17}	0.01	0.04	0	0.0285	0.0285	0	0	0	0	0	0	0	0	0	0	0	0	0	0	0	0
F_{18}	0	0	0	0.04	0.0225	0	0.0255	0.0115	0	0	0	0	0	0	0	0	0	0	0	0.01	0.01
F_{19}	0.01	0	0	0	0	0	0	0.0115	0.0121	0	0	0	0	0	0.0102	0	0.01	0	0	0.01	0

F_{20}	0	0	0	0	0	0.04	0.04	0.0225	0.01	0.01	0	0	0	0	0	0	0	0	0	0	0
F_{21}	0	0	0	0	0	0	0	0	0	0	0	0.01	0.01	0.01	0	0	0	0	0	0	0

Table S4. The overall effect matrix of the representative risk factors of gas explosions.

	F_1	F_2	F_3	F_4	F_5	F_6	F_7	F_8	F_9	F_{10}	F_{11}	F_{12}	F_{13}	F_{14}	F_{15}	F_{16}	F_{17}	F_{18}	F_{19}	F_{20}	F_{21}
F_1	1	0	0	0.01	0.01	0	0.01	0	0.01	0.01	0	0	0	0	0	0	0	0	0	0	0.01
F_2	0	1	0	0.0225	0.0225	0.01	0	0	0	0	0	0	0	0	0	0	0	0	0	0	0.01
F_3	0.01	0.01	1	0	0	0	0	0	0	0	0	0	0	0	0	0	0	0	0	0	0
F_4	0	0	0	1	0	0	0	0	0	0	0	0	0	0	0	0	0	0	0	0	0
F_5	0	0	0	0	1	0	0	0	0	0	0	0	0	0	0	0	0	0	0	0	0
F_6	0	0	0	0	0	1	0	0	0	0	0	0.0225	0	0.01	0.01	0	0	0	0	0	0
F_7	0	0	0	0	0	0	1	0	0	0	0	0	0	0	0	0	0	0	0	0	0
F_8	0	0	0	0	0	0	0	1	0	0	0	0.01	0.01	0.01	0	0	0	0	0	0	0
F_9	0	0	0	0	0	0	0	0	1	0	0	0.01	0.01	0.01	0	0	0	0	0	0	0
F_{10}	0	0	0	0	0	0	0	0	0	1	0	0	0	0	0	0	0	0	0	0	0
F_{11}	0	0	0	0	0	0	0	0	0	0.01	1	0.01	0.04	0.0225	0.01	0	0	0	0	0	0
F_{12}	0	0	0	0	0	0	0	0	0	0	0	1	0	0	0	0	0	0	0	0	0
F_{13}	0	0	0	0	0	0	0	0	0	0	0	0	1	0	0	0	0	0	0	0	0
F_{14}	0	0	0	0	0	0	0	0	0	0	0	0	0	1	0	0	0	0	0	0	0
F_{15}	0	0	0	0	0	0	0	0	0	0	0	0	0	0	1	0	0	0	0	0	0
F_{16}	0	0.04	0	0.05	0.049	0.0303	0	0	0	0	0	0	0	0	0	1	0	0.01	0	0.024	0
F_{17}	0.01	0.04	0	0.0285	0.0285	0	0	0	0	0	0	0	0	0	0	0	1	0	0	0	0
F_{18}	0	0	0	0.04	0.0225	0	0.0255	0.0115	0	0	0	0	0	0	0	0	0	1	0	0.01	0.01
F_{19}	0.01	0	0	0	0	0	0	0.0115	0.0121	0	0	0	0	0	0.0102	0	0.01	0	1	0.01	0
F_{20}	0	0	0	0	0	0.04	0.04	0.0225	0.01	0.01	0	0	0	0	0	0	0	0	0	1	0
F_{21}	0	0	0	0	0	0	0	0	0	0	0	0.01	0.01	0.01	0	0	0	0	0	0	1

Table S5. The reachability matrix of the representative risk factors of gas explosions.

	F_1	F_2	F_3	F_4	F_5	F_6	F_7	F_8	F_9	F_{10}	F_{11}	F_{12}	F_{13}	F_{14}	F_{15}	F_{16}	F_{17}	F_{18}	F_{19}	F_{20}	F_{21}	*Driving Power*

*F*1	1	0	0	1	1	0	1	0	1	1	0	0	0	0	0	0	0	0	0	0	1	7
*F*2	0	1	0	1	1	1	0	0	0	0	0	0	0	0	0	0	0	0	0	0	1	5
*F*3	1	1	1	0	0	0	0	0	0	0	0	0	0	0	0	0	0	0	0	0	0	3
*F*4	0	0	0	1	0	0	0	0	0	0	0	0	0	0	0	0	0	0	0	0	0	1
*F*5	0	0	0	0	1	0	0	0	0	0	0	0	0	0	0	0	0	0	0	0	0	1
*F*6	0	0	0	0	0	1	0	0	0	0	0	1	0	1	1	0	0	0	0	0	0	4
*F*7	0	0	0	0	0	0	1	0	0	0	0	0	0	0	0	0	0	0	0	0	0	1
*F*8	0	0	0	0	0	0	0	1	0	0	0	1	1	1	0	0	0	0	0	0	0	4
*F*9	0	0	0	0	0	0	0	0	1	0	0	1	1	1	0	0	0	0	0	0	0	4
*F*10	0	0	0	0	0	0	0	0	0	1	0	0	0	0	0	0	0	0	0	0	0	1
*F*11	0	0	0	0	0	0	0	0	0	1	1	1	1	1	1	0	0	0	0	0	0	6
*F*12	0	0	0	0	0	0	0	0	0	0	0	1	0	0	0	0	0	0	0	0	0	1
*F*13	0	0	0	0	0	0	0	0	0	0	0	0	1	0	0	0	0	0	0	0	0	1
*F*14	0	0	0	0	0	0	0	0	0	0	0	0	0	1	0	0	0	0	0	0	0	1
*F*15	0	0	0	0	0	0	0	0	0	0	0	0	0	0	1	0	0	0	0	0	0	1
*F*16	0	1	0	1	1	1	0	0	0	0	0	0	0	0	0	1	0	1	0	1	0	7
*F*17	1	1	0	1	1	0	0	0	0	0	0	0	0	0	0	0	1	0	0	0	0	5
*F*18	0	0	0	1	1	0	1	1	0	0	0	0	0	0	0	0	0	1	0	1	1	7
*F*19	1	0	0	0	0	0	0	1	1	0	0	0	0	0	1	0	1	0	1	1	0	7
*F*20	0	0	0	0	0	1	1	1	1	1	0	0	0	0	0	0	0	0	0	1	0	6
*F*21	0	0	0	0	0	0	0	0	0	0	0	1	1	1	0	0	0	0	0	0	1	4
Dependence Power	4	4	1	6	6	4	4	4	4	4	1	6	5	6	4	1	2	2	1	4	4	

Table S6. The results of the level partitions of the reachability matrix Iteration I to Iteration IV.

Representative risk factors	Reachability set	Antecedent set	Intersection set	Level
F_1	F_1, F_4, F_5, F_7, F_9, F_{10}, F_{21}	F_1, F_3, F_{17}, F_{19}	F_1	III
F_2	F_2, F_4, F_5, F_6, F_{21}	F_2, F_3, F_{16}, F_{17}	F_2	III
F_3	F_1, F_2, F_3	F_3,	F_3	IV
F_4	F_4	F_1, F_2, F_4, F_{16}, F_{17}, F_{18}	F_4	I

F_5	F_5	F_1, F_2, F_5, F_{16}, F_{17}, F_{18}	F_5	I
F_6	F_6, F_{12}, F_{14}, F_{15}	F_2, F_6, F_{16}, F_{20}	F_6	II
F_7	F_7	F_1, F_7, F_{18}, F_{20}	F_7	I
F_8	F_8, F_{12}, F_{13}, F_{14}	F_8, F_{18}, F_{19}, F_{20}	F_8	II
F_9	F_9, F_{12}, F_{13}, F_{14}	F_1, F_9, F_{19}, F_{20}	F_9	II
F_{10}	F_{10}	F_1, F_{10}, F_{11}, F_{20}	F_{10}	I
F_{11}	F_{10}, F_{11}, F_{12}, F_{13}, F_{14}, F_{15}	F_{11}	F_{11}	II
F_{12}	F_{12}	F_6, F_8, F_9, F_{11}, F_{12}, F_{21}	F_{12}	I
F_{13}	F_{13}	F_8, F_9, F_{11}, F_{13}, F_{21}	F_{13}	I
F_{14}	F_{14}	F_6, F_8, F_9, F_{11}, F_{14}, F_{21}	F_{14}	I
F_{15}	F_{15}	F_6, F_{11}, F_{15}, F_{19}	F_{15}	I
F_{16}	F_2, F_4, F_5, F_6, F_{16}, F_{18}, F_{20}	F_{16},	F_{16}	V
F_{17}	F_1, F_2, F_4, F_5, F_{17}	F_{17}, F_{19}	F_{17}	IV
F_{18}	F_4, F_5, F_7, F_8, F_{18}, F_{20}, F_{21}	F_{16}, F_{18}	F_{18}	IV
F_{19}	F_1, F_8, F_9, F_{15}, F_{17}, F_{19}, F_{20}	F_{19}	F_{19}	V
F_{20}	F_6, F_7, F_8, F_9, F_{10}, F_{20}	F_{16}, F_{18}, F_{19}, F_{20}	F_{20}	III
F_{21}	F_{12}, F_{13}, F_{14}, F_{21}	F_1, F_2, F_{18}, F_{21}	F_{21}	II
